# Supplementary material for: Studies on chain shuttling polymerization reaction of nonbridged half-titanocene and bis(phenoxy-imine) Zr binary catalyst system
Source: R Soc Open Sci. 2019 Apr 10;6(4):182007. doi: 10.1098/rsos.182007 (PMC6502386; doi:10.1098/rsos.182007)
Supplement: Figures S1 - S7 [file rsos182007supp1.docx]

**Royal Society Open Science**

**Electronic Supplementary Material**

**Studies on chain shuttling polymerization reaction of nonbridged**

**half-titanocene and bis(phenoxy-imine) Zr binary catalyst system**

**Qinwen Xu ^1,2,^, Rong Gao ^1,2,^ and Dongbing Liu*^1,3^**

^1^ Polyoleﬁns National Engineering and Research Center, Sinopec Beijing Research Institute of Chemical Industry, Beijing 100013, PR China

^2^ Polyethylene Research Center, Sinopec Beijing Research Institute of Chemical Industry, Beijing 100013, PR China

^3^ Institute of Catalysis Science, Sinopec Beijing Research Institute of Chemical Industry, Beijing 100013, PR China

* Author for Correspondence: liudb.bjhy@sinopec.com; Present address: Institute of Catalysis Science, Sinopec Beijing Research Institute of Chemical Industry, Beijing 100013, PR China





**Figure S1.** The effect of the amount of ZnEt_2_ on the melting point and melting enthalpy of ethylene polymers obtained with the dual-catalyst system


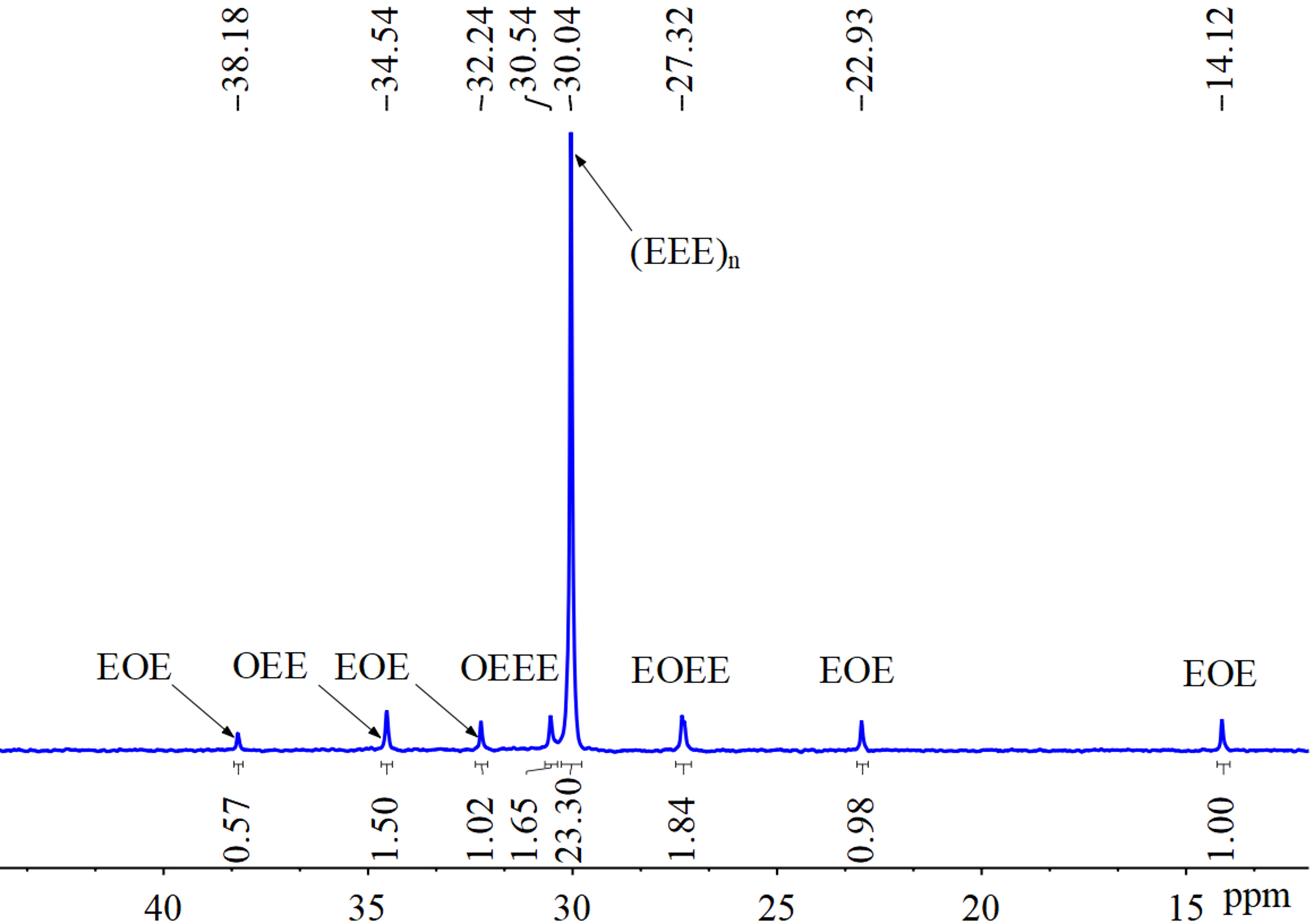


**Figure S2.** ^13^C NMR spectra of the polymers (Run 9) at 100 ℃


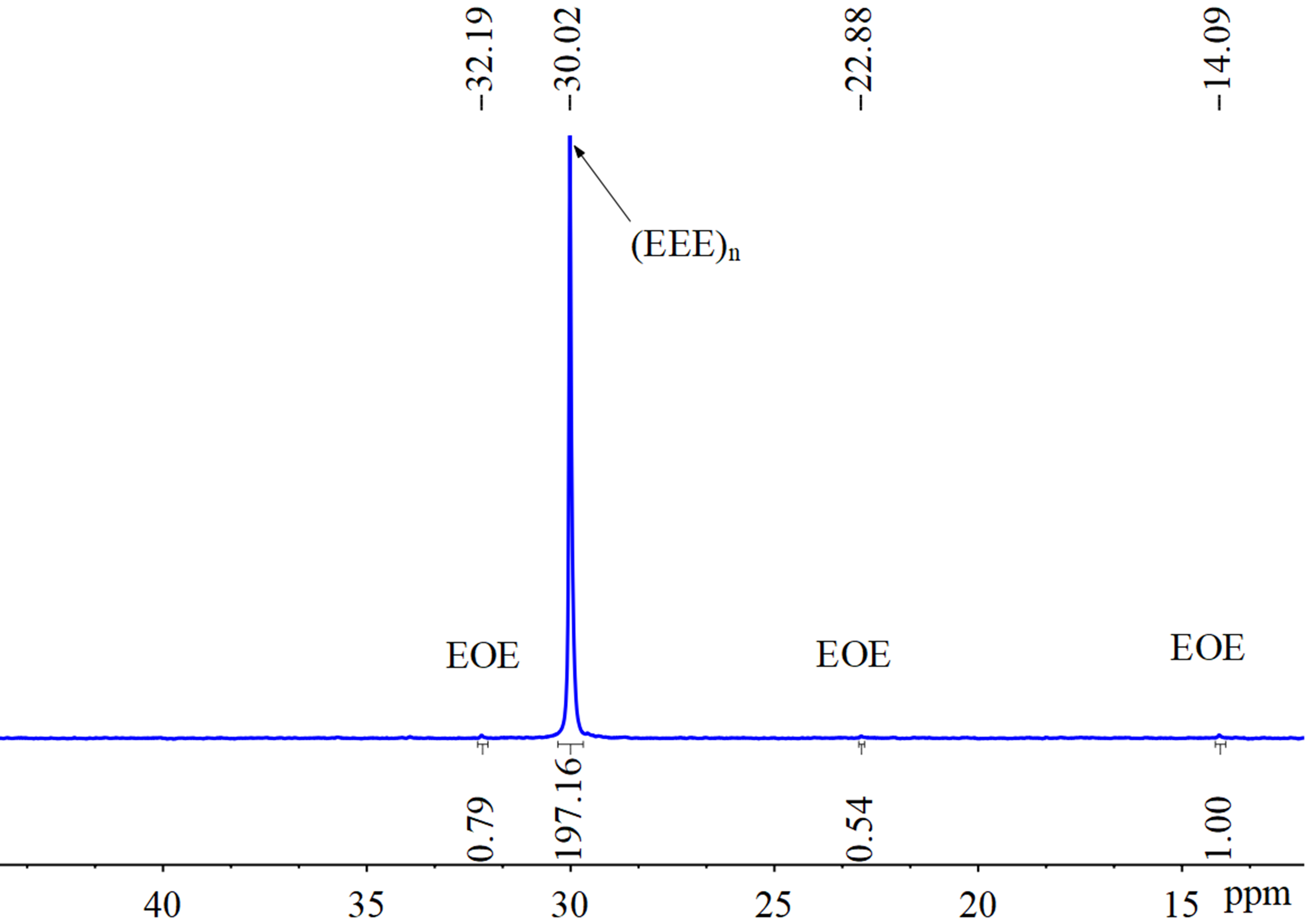


**Figure S3.** ^13^C NMR spectra of the polymers (Run 11) at 100 ℃


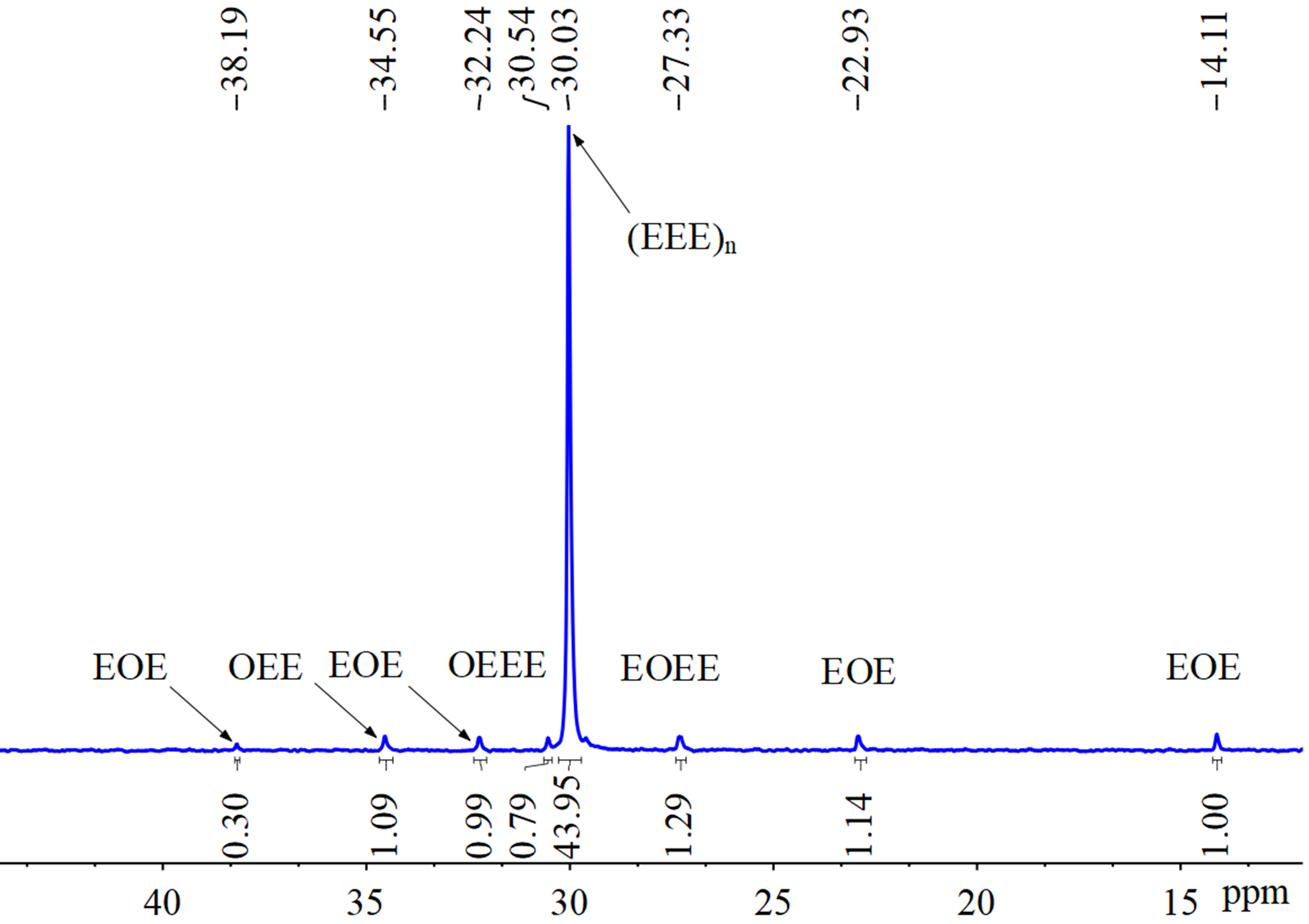


**Figure S4.** ^13^C NMR spectra of the polymers (Run 14) at 100 ℃





**Figure S5.** The effect of the 1-octene on the molecular weight and polydispersity of polymers obtained with individual catalysts





**Figure S6.** The effect of the 1-octene on the melting point and melting enthalpy of polymers obtained with individual catalysts





**Figure S7.** The effect of ZnEt_2_ on the melting point and melting enthalpy of copolymers obtained with individual catalysts
